# Bibliographical Notices

**Published:** 1856-10

**Authors:** 


### BIBLIOGRAPHICAL NOTICES.

*The Sydenham Society.*—We deem it our duty to lay before our readers all facts which may promote their best interests, and aid in answering their wishes for the cultivation of their scientific knowledge.

The Society above named is developing and actively dispensing a vast fund of interesting and useful matter, and those who can, should at once subscribe. These annual contributions are most valuable, and in this way are obtained at much lower rates than by private purchase.

There seems to us to be an omission in the circular which we publish below entire, and that is that Prof. Dunglison does not intimate how the works are to be distributed to subscribers.

"*The Sydenham Society, of London.*—This Society was instituted in 1843, with the view of supplying its members with Standard Medical Works. The subscription, constituting a member, is five dollars annually, payable in advance. The following extracts from the laws will explain the objects of the Society:—

I. The Society is instituted for the purpose of meeting certain acknowledged deficiencies in existing means for diffusing medical literature, which are not likely to be supplied by the efforts of individuals, and shall be called the "Sydenham Society."

II. The Society will carry its objects into effect by a succession of publications, embracing, among others: 1. Reprints of standard English works, which are rare or expensive; 2. Miscellaneous selections from the ancient and from the earlier modern authors, reprinted or translated; 3. Digests of the old and voluminous authors, British and foreign, with occasional biographical and bibliographical notices; 4. Translations of the Greek and Latin medical authors, and of works in the Arabic and other Eastern tongues, accompanied, when it is thought desirable, by the original text; 5. Translations of recent foreign works of merit; 6. Original works of merit, which might prove valuable as books of reference, but which would not otherwise be published, from the slender chance of their meeting with a remunerating sale—such as bibliographies, alphabetical and digested indexes to voluminous periodical publications, &c.

Three volumes, handsomely bound in a uniform manner in cloth, gilt edged, are usually issued in the year.

LIST OF THE SOCIETY'S WORKS, of which copies are still on hand, and from which new members, subscribing for the current year, may make a selection, on payment ot an additional five dollars for any three volumes, with the exception of those to which an asterisk is affixed. Those to which an asterisk is affixed, or any other single volume, may be had for $2,50 per volume.

Sydenhami Opera Omnia; 1 vol. Hasse's Pathological Anatomy; 1 vol. Rhazes on the Smallpox and Measles; 1 vol. The Works of Hewson; portrait and plates; 1 vol. Dupuytren's Lectures on Diseases and Injuries of Bones; 1 vol. Dupuytren on Lesions of the Vascular System, &c.; 1 vol. Memoirs of the French Academy of Surgery; 1 vol. Feuchtersleben's Medical Psychology; 1 vol. Microscopical Researches of Schwann and Schleiden; 1 vol; Plates. The Works of W. Harvey, M. D.; 1 vol. The Genuine Works of Hippocrates; 2 vols. Essays on Puerperal Fever, and other Diseases peculiar to Women; 1 vol. The Works of Sydenham, translated from the Latin; 2 vols. Unzer and Prochaska on the Nervous System; 1 vol. Annals of Influenza; 1 vol. Romberg on Diseases of the Nervous System; 2 vols. Kolliker's Manual of Human Histology; 2 vols; Woodcuts. *Rokitansky's Pathological Anatomy; Complete in 4 vols. *Hunter on the Gravid Uterus; 1 vol; folio; 34 plates, with descriptive letter-press. Wedl's Pathological Histology; 1 vol; Woodcuts. Oesterlen's Medical Logic; 1 vol. Velpeau on Diseases of the Breast; 1 vol. The Works of Aretæus, Greek and English; 1 vol.

RICHARD J. DUNGLISON, M. D.,
*Hon. Local Secretary for Philadelphia.*

*Hand-book of Organic Chemistry: for the use of Students.* By William Gregory, M. D. F. R. S. E., Prof. of Chemistry in the University of Edinburgh. Edited by J. Milton Sanders, M. D. L. L. D.

The fourth American, from the fourth London edition, has reached us from the publishers, A. S. Barnes & Co., N. Y.; octavo, pp. 471: cloth, 1857.

While "of the making of books there is no end," it is really gratifying to know that among the multiplicity of them, we are able to find so many truly valuable. That Prof. Gregory should put forth a work of high worth upon Chemistry, is however, only what would be expecteed from his known erudition and skill in this branch of knowledge, and he has accomplished it in the work before us, which is intended for the use of students. It is intended to accompany a work on Inorganic Chemistry, by the same author, which we noticed in a preceding number of the Journal. In looking through this work, we are led, in view of the comparatively recent date of the first efforts towards the development of the science of Organic Chemistry, to express astonishment, almost, at the immense amount of knowledge which has been gathered, and the laborious research which it has necessitated for its acquisition.

It is scarcely a score of years since the attention of scientific men has been directed, to any considerable extent, to this field, and yet the science is nearly as complete to-day as any other of the natural sciences. And if Liebig may have the credit of being the man to awaken the scientific world to its importance to the welfare of all classes of society and in most of the useful arts of civilized life, it is but justice to acknowledge that in the labor of developing it, to an extent to make it of practical utility, such men as Dr. Gregory have performed fully their share.

In medicine, while we are free to acknowledge that Organic Chemistry has done much less than it has seemed to promise, yet in other departments of life, it has proven itself competent to the accomplishment of much more. And in medicine, even, it is so far from established that it is incapable of effecting what has been expected of it, that we are led to the persuasion that it fails only because of inability, with our present knowledge, properly to compute those modifying and disturbing influences resulting from the action of vital force upon chemical affinities.

Of one thing we feel assured, that in the routine of medical studies, chemistry has heretofore, and does now, come in for too small a share of the student's time and attention, and this the more, since to a large proportion of the profession, it is a branch to which opportunity and the active duties of practice make it nearly impossible to give large practical study. Even in Inorganic Chemistry, there is by far too little known by the mass of the profession, and more than once within our own observation, most dangerous results have occurred in practice.

But it is not alone the medical profession which is interested in the results of researches in chemical science. Perhaps the agriculturist is to a greater extent interested than any other, and it is said that in England so great a change has been effected in the modes of agriculture and in the prosecution of many of the arts, through the influence of chemistry in its different departments, as to be no less a revolution than if the form of government had been changed. The people are changed because of the change in the character of their necessities, and these are all for the better.

And this revolution is brought about by the efforts of such men as Prof. Gregory, who, by the prosecution of a practical science, bring home to the people the means of working out for themselves great personal and public benefits.

With us, in the West, at least, we have not yet come to feel the necessity of such knowledge any farther than within the medical profession, but it is time that, in the educational training of our youth, we make this an important item. And Chemistry should be taught, not only in our colleges and academies, but in our common schools, and in each and all of these it should be taught experimentally and practically. Not that we expect the time to come when all will be chemists in the sense in which that term is now used, but they should understand well its principles, and to this, it is necessary to teach, to a great extent, by the inductive method. The work before us, is well adapted to the use of academies and colleges and to common schools even, after the student is somewhat advanced. As a work of reference it is valuable, but its index is exceedingly defective as to convenience. We may speak more at length hereafter of this work; at present we have been induced to speak rather of the importance of that of which it treats than to give a review of the work itself, which, indeed, would be impossible, except upon more than the cursory examination we have as yet been able to give it.

